# pELMO, an optimised in-house cloning vector

**DOI:** 10.1186/s13568-017-0324-2

**Published:** 2017-01-24

**Authors:** Andrea E. Ramos, Marina Muñoz, Darwin A. Moreno-Pérez, Manuel A. Patarroyo

**Affiliations:** 1 Molecular Biology and Immunology Department, Fundación Instituto de Inmunología de Colombia, Cra. 50 # 26-20, Bogotá, Colombia; 2 PhD Programme in Biomedical and Biological Sciences, Universidad del Rosario, Bogotá, Colombia; 3 School of Medicine and Health Sciences, Universidad del Rosario, Bogotá, Colombia

**Keywords:** Recombinant DNA technology, Cloning vector, *ccdB* killer gene, Blunt-ended, PCR cloning

## Abstract

DNA cloning is an essential tool regarding DNA recombinant technology as it allows the replication of foreign DNA fragments within a cell. pELMO was here constructed as an in-house cloning vector for rapid and low-cost PCR product propagation; it is an optimally designed vector containing the *ccdB* killer gene from the pDONR 221 plasmid, cloned into the pUC18 vector’s multiple cloning site (Thermo Scientific). The *ccdB* killer gene has a cleavage site (CCC/GGG) for the *Sma*I restriction enzyme which is used for vector linearisation and cloning blunt-ended products. pELMO transformation efficiency was evaluated with different sized inserts and its cloning efficiency was compared to that of the pGEM-T Easy commercial vector. The highest pELMO transformation efficiency was observed for ~500 bp DNA fragments; pELMO vector had higher cloning efficiency for all insert sizes tested. In-house and commercial vector cloned insert reads after sequencing were similar thus highlighting that sequencing primers were designed and localised appropriately. pELMO is thus proposed as a practical alternative for in-house cloning of PCR products in molecular biology laboratories.

## Introduction

Cloning polymerase chain reaction (PCR) products into plasmid vectors is a common practice in a molecular biology research laboratory. Cloning systems are characterised by allowing PCR product incorporation (Bernard 1996) into a circular plasmid; this can be further propagated to obtain an appropriate number of DNA copies whose integrity can be verified by sequencing and may be cryopreserved for future use (Gruber et al. 2008). Numerous different sized cloning vectors are available having several cloning sites, many being exclusive to the bacterial strains and culture media required for their propagation. Such features involve constraints, thereby leading to variable transformation efficiency (Phillips et al. 2000). Simple strategies have been developed nowadays for constructing, modifying and propagating cloning vectors without the need for special equipment, reagents or having advanced cloning knowledge (Ma et al. 2014; Weibel et al. 2013). Such approaches are an alternative for optimising resources in molecular biology research since the currently available commercial PCR-DNA cloning kits are expensive and involve using of additional reagents for the growth and identification of recombinant colonies (Cheong et al. 2009, 2012).

Thymine and adenine (TA) cloning vectors have been one of the most commonly used plasmids for achieving high cloning efficiency (Holton and Graham 1991; Ito et al. 2000). However, they require adenine nucleotides to be added at the insert’s 5′ and 3′ ends, this can be time-consuming when ligating the blunt ends of fragments amplified by proof-reading DNA polymerases (blunt-end ligation is regarded as low-efficient, involving the risk of plasmid re-circularisation). The former increases the consumption of time and money for laboratories constantly working on cloning (Guo and Bi 2002). Cloning recombinant bacteria by means of *ccdB* (coupled cell division *B* gene) gene selection offers an advantage over *E. coli* clones selected via the *LacZ* system via blue/white screening (Bernard 1996; Messing et al. 1977). The *ccdB* gene product obstructs DNA gyrase activity, inducing GyrA-DNA complex formation promoting plasmid and chromosomal DNA rupture. This causes cell non-viability, due to death in most *E. coli* strains (Bernard 1996). *ccdB* activity can be inhibited in two ways: expression of the *ccdA* gene-encoded product or *ccdB* sequence disruption through DNA fragment insertion into its already-embedded multiple cloning site (Maki et al. 1992). Regarding the latter, transformants only bear the recombinant plasmid (the disrupted *ccdB* sequence will thus survive and grow). This saves a lot of time when choosing candidates for colony PCR screening.

This paper deals with an optimisation of the home-made cloning vector pUC18/*ccdB* by constructing pELMO, an optimised blunt-end vector having better cloning efficiency than TA commercial cloning vector pGEM-T Easy. This plasmid is intended to be used for efficient, fast and cheap cloning of variable sized PCR products.

## Materials and methods

### Primer design

Gene Runner software was used for designing two sets of primers to be used in pELMO construction. The first set flanked the *ccdB* gene sequence in the pDONR221 vector (Invitrogen Corp., CA, USA) (*ccdB*-ecoRI: 5′-CG**G/AATTC**AAGCCAGATAACAGTATGCG-3′ and *ccdB*-pstI: 5′-AAG**CTGCA/G**ACTGGCTGTGTAT-3′) whilst the second targeted regions 50 mer upstream and downstream of the *Sma*I enzyme cleavage site located in the *ccdB* sequence. (ccdBsec-F:5′-TGCAGTTTAAGGTTTACACC-3′/ccdBsec-R:5′-CACCACCGGGTAAAGTTC- 3′) (Table 1). The latter primer set was used for sequencing the genes of interest after cloning in the vector restriction site. *ccdB*-ecoRI and *ccdB*-pstI primers represented added restriction sites for *Eco*RI and *Pst*I enzymes (in bold) at their 5′ end with a couple of stabilising nucleotides, following New England BioLabs’ guidelines (New-England-Biolabs 2000).

**Table 1:** Primer sequences used in this study

Target	Locus (access number)	Primer type	Name	Primer sequence 5′→3′ (added restriction site is underlined)	Melting temperature (°C)	Expected size (bp)
pDONR-221	ccdB (U51588.1)	Encoding gene/colony PCR primer	ccdB-ecoRI	CGg/aattcAAGCCAGATAACAGTATGCG	60	675
			ccdB-pstI	AActgca/gACTGGCTGTGTAT		
pELMO	ccdB (U51588.2)	Sequencing primer	ccdBsec-F	TGCAGTTTAAGGTTTACACC	56	161 bp+ (DNA insert)
			ccdBsec-R	CACCACCGGGTAAAGTTC		
*Plasmodium falciparum*	CSP (XM_001351086)	Encoding gene/colony PCR primer	F-NCOI	c/catggAGTGCTATGGAAGTTCGT	62	246
			R-XHO	CCGc/tcgagTCATGCATTTGGATCAGGATTAC		
*Plasmodium falciparum*	MSP-1(XM_001352134)	Encoding gene/colony PCR primer	F-CT	CATGc/catggTAGTTGTATTACCCATTTTT	55	512
			R-CT	CCGc/tcgagTCAGATAACTTTTTTAATTGATTC		
*Plasmodium falciparum*	EBA-175 (XM_001349171)	Encoding gene/colony PCR primer	F-NCO	CATGc/catggTATCCACTAAAGATGTATGTG	54	891
			EBARII-Stop	CCGc/tcgagTCATCCATCCGTACGAGTTTC		
*Neospora caninum*	Nc5 (AY459289.1)	Encoding gene/colony PCR primer	Np21+	CCCAGTGCGTCCAATCCTGTAGAC	60	350
			Np6+	CTCGCCAGTCAACCTACGTCTTCT		
*Plasmodium vivax*	ARNP (Pv_Sal1_chr10)-(828,231–828,827)	Encoding gene/colony PCR primer	PvARNP-D	ATGAAAAAAGTGGCCTCGTT	54	597
			PvARNP-R	AAGGTTGAAGAAAAATTTAAAAA		
*Plasmodium vivax*	PvRON4 (KF378614)	Encoding gene primer	pvron4dir	CACAGTGCAACCATGTCTCG	68	~2300
			pvron4rev	GCAAGCTAATTTCACAAGTCTTC		
*Plasmodium vivax*	PvRON4 (KF378614)	Colony PCR primer	pvron4intdir	CACAGTGCAACCATGTCTCG	60	844
			pvron4intrev	GCAAGCTAATTTCACAAGTCTTC		
pGEM-T easy vector		Sequencing primer	SP6	ATTTAGGTGACACTATAG	54	177 bp+ (DNA insert)
			T7	AATACGACTCACTATAG		

The features for each PCR primer set used in this study

### Obtaining the ccdB gene

The pDONR221 vector (Invitrogen Corp., CA, USA) was first propagated in One Shot *ccdB* Survival 2 T1R cells, a *ccdB* action resistant strain, and then isolated using an UltraClean 6 Minute Mini Plasmid Prep Kit (MO BIO Laboratories, Inc). This was then used as template for PCR amplification of *ccdB* 659 base pairs (bp) by means of ccdB-ecoRI and ccdB-pstI primers. This gene is located in pDONR221 nucleotide positions 1163–1821 (5′→3′). KAPA HiFi HotStart Readymix (Kapa Biosystems) was used for amplification. The reaction mixture consisted of 0.3 μM of each primer in final 25 μL volume. The thermic profile was as follows: initial denaturing step at 95 °C for 5 min, followed by 35 cycles consisting each of 20 s denaturing at 98 °C, 20 s annealing at 56 °C and 1 min extension step at 72 °C, followed by a final extension step at 72 °C for 5 min. PCR product was then purified by Wizard SV Gel and PCR Clean-Up System (Promega, WI, USA), following the manufacturer’s instructions. The amplicon was then sequenced using a BigDye Terminator kit (Macrogen, Seoul, South Korea) with *ccdB*-ecoRI and *ccdB*-pstI primers (Table 1). The resulting sequences were analysed to verify the absence of mutations and reading frame conservation. The PCR product was quantified and digested after *ccdB* verification, first with *Eco*RI and then with *Pst*I restriction enzymes (New England Biolabs, Herts, UK). Enzymatic digestion was performed in 50 μL volume, containing: 1× NEBuffer 3.1, 0.1 U *Eco*RI and 10 μL purified *ccdB* PCR-product. Following inactivation at 65 °C for 20 min, the following were added: 1× NEBuffer 3.1, 10 μg/mL BSA and 0.5 U *Pst*I. The reaction was inactivated at 80 °C for 20 min and stored at −20 °C until use.

### pUC18 manipulation

Vector pUC18 was cloned in One Shot TOP10 chemically competent *E. coli* (Invitrogen) and purified using an UltraClean 6 Minute Mini Plasmid Prep Kit (MO BIO). The isolated product was digested with *Eco*RI and *Pst*I enzymes, as above; corresponding restriction sites flanked the pUC18 multiple cloning site (MCS). Digested products were then purified on low melting point agarose gels by the Wizard SV Gel and PCR Clean-Up System (Promega).

### pELMO construction

**Fig. 1:**
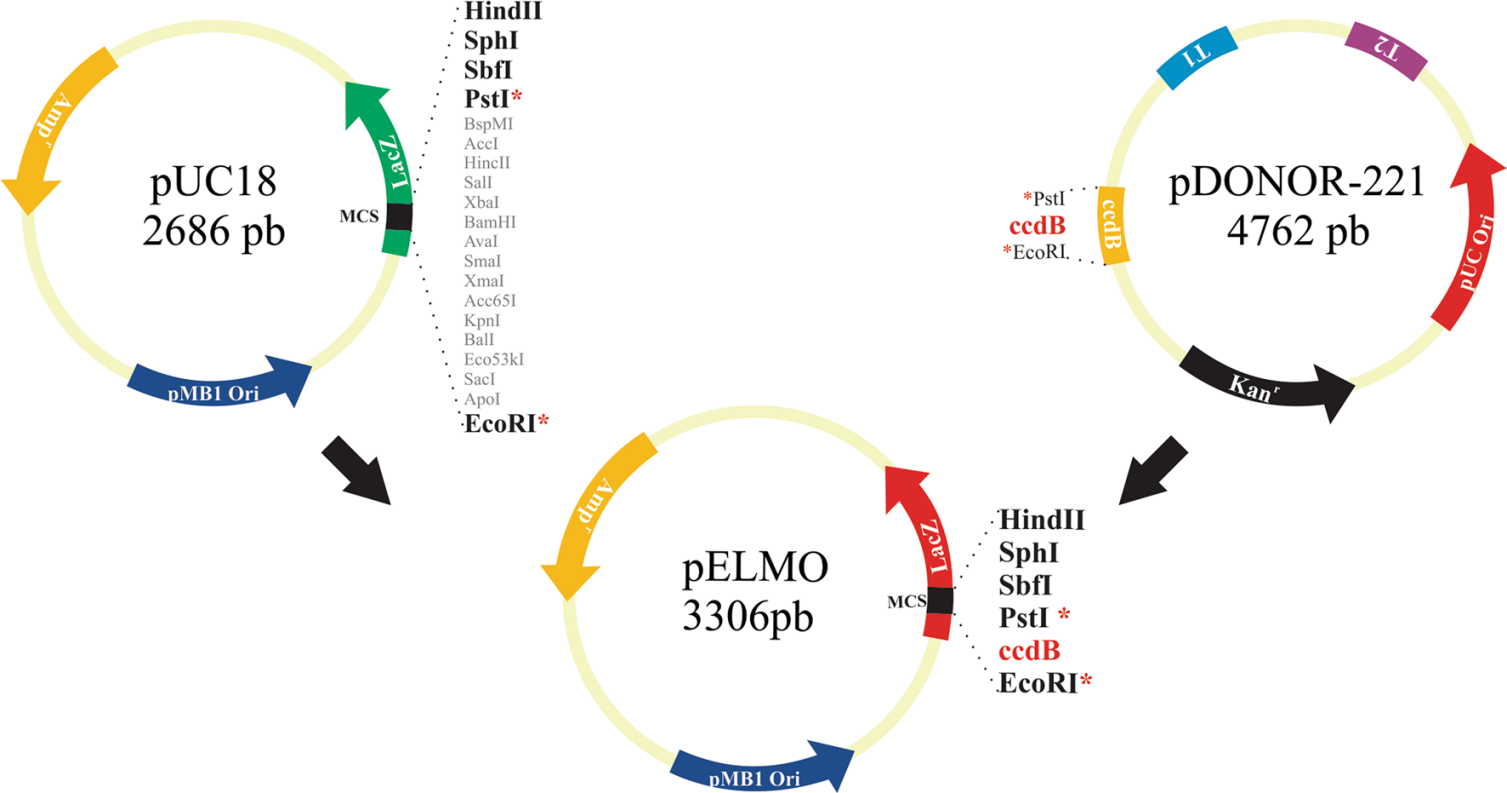
Overview of pELMO vector construction. Construction details are provided in the text. *Red asterisks* indicate *Eco*RI/*Pst*I recognition sites used for pELMO construction. The *ccdB* gene cloned between *Eco*RI and *Pst*I restriction sites was amplified from pDONOR-221 vector

The previously purified and digested *ccdB* product was then ligated to digested pUC18, thus yielding the pELMO vector. Rapid DNA Ligation Kit #K1422 (Thermo Fisher Scientific, Inc) was used for ligation. The reaction mixture consisted of 100 ng digested pUC18 vector, *ccdB* insert PCR-DNA (at 1:1 molar ratio), 1× Rapid ligation Buffer and 1 μL T4 DNA ligase in a total volume of 10 μL. Such mixture was incubated for 4 h at 22 °C. Figure 1 summarises the pELMO construction strategy. The pELMO construct was propagated on One Shot *ccdB* survival cells (Invitrogen).

### pELMO transformation efficiency

DNA fragments from *csp* (246 bp) and *msp1* (512 bp) genes were amplified, in addition to the *Plasmodium falciparum* 3D7 strain *eba*-175 region II (891 bp) to observe transformation efficiency in pELMO vector for different sized inserts through direct cloning. Such amplification was performed through high fidelity DNA polymerase (Kapa Biosystems), using the corresponding primer sets from Table 1. All PCR products were cleaned using Wizard SV Gel and PCR Clean-Up System (Promega) before T4 ligation reaction (Promega). 50 ng linearised pELMO were used in ligation reaction, along with 50 ng of each purified PCR product. Reactions were incubated for 12 h at 18 °C with T4 DNA ligase, according to the manufacturer’s recommendations. Subsequent *E. coli* TOP10 transformation involved: 100 μL *E. coli* TOP10 chemically competent cells (Invitrogen) incubated with 5 μL ligation mixture for 20 min on ice, heat shocked at 42 °C for 2 min and propagated in SOC medium at 37 °C, with shaking for 1 h. Transformed cells were spun at 10,000 rpm and then homogenised in 100 μL SOC medium. Later, 80 μL suspended cells were plated on Luria–Bertani (LB)-agar plates containing 100 μg/mL ampicillin (amp). The colonies were counted and analysed by colony PCR after 16 h; products were then confirmed by 1% agarose gel electrophoresis. The Bacteria Transformation Efficiency Calculator (http://www.sciencegateway.org/tools/transform.htm) was used for calculating transformation efficiency for each PCR product ligated into pELMO and expressed as transformants/µg plasmid.

### Comparing pELMO cloning efficiency with that of a commercial vector

*Neospora caninum nc5* gene (350 bp) and *Plasmodium vivax* apical rhoptry neck protein (*arnp*-597 bp) and rhoptry neck protein 4 gene (*ron4*-2.3 kb) PCR amplification products were cloned in pGEM-T Easy (Promega) system and pELMO to compare their cloning efficiency (Table 1 lists the corresponding primers). The KAPA HiFi Ready Mix system was used for amplifying inserts, using the following thermal profile: initial denaturation at 95 °C for 5 min, followed by 35 cycles each of 98 °C for 30 s, the corresponding primer’s T_m_ (Table 1) for 20 s, 72 °C for 2 min and a final extension of 72 °C for 5 min. PCR products were ligated into pELMO after purification, as shown before, and transformed into TOP10 chemically competent *E. coli* cells (Invitrogen).

Alternatively, each aforementioned insert was subjected to 3′ end-adenine addition through Taq polymerase (Bioline) for ligation at pGEM-T Easy vector multiple cloning site (Promega, WI, USA). Ligations were performed as recommended by the manufacturer to improve clone recovery (3:1 insert-to-vector ratio) (Litterer 2009). The resulting constructs were transformed in *E. coli* JM109 competent cells (Promega). Clones from each plate were randomly chosen and analysed by colony PCR after 15 h incubation at 37 °C with Np21+/Np6+, PvARNP-D/PvARNP-R and Pvron4intdir/Pvron4intrev primer sets (Table 1). PCR reactions were performed in a 10 μL volume containing 1X Green GoTaq reaction, 0.5 µM each primer, 1.5 mM MgCl_2_, 0.2 mM of each dNTP and 0.6 U GoTaq DNA polymerase (Promega). PCR conditions were as follows: 95 °C for 5 min, and 35 cycles each of 94 °C for 30 s, the corresponding primer’s T_m_ (Table 1) for 15 s and 72 °C, 30 s. Final extension was 72 °C for 5 min. An UltraClean mini plasmid prep purification kit (MO BIO) was used for growing the recombinant clones and purifying the plasmid. Cloning efficiency was calculated as the ratio of positive colonies by PCR over the total amount of colonies on each LB-amp plate. Plasmid identity was confirmed by sequencing, using the ccdBsec-F/ccdBsec-R and SP6/T7 primer sets; these were then aligned with the corresponding pELMO and pGEM-T Easy cloning site flanking sequences.

### Statistical analysis

pELMO transformation efficiency was reported as the amount of transformants per µg plasmid. Frequency, mean values and standard deviation (SD) were calculated from the measurements of two independent experiments. Cloning efficiency was measured as the percentage of the amount of colonies confirmed positive by PCR for the insert of interest regarding the total of colonies tested. Each vector’s transformed colonies were regarded as independent populations. Statistical significance was assessed by comparing means. Cloning event frequency was reported with corresponding 95% confidence intervals (CI), estimated by the Bootstrap method. Percentage cloned insert length obtained through sequencing was also compared regarding both vectors. Statistical significance was inferred as mentioned above. STATA software package 11.0 (Stata Corporation, College Station, TX) was used for all statistical analysis.

## Results

### Constructing the pELMO positive-selection cloning vector

The in-house vector presented here was constructed by combining commercial pUC18 and the pDONR-221 plasmid’s *ccdB* gene encoding region; toxic gene system sequence integrity was verified by sequencing, corresponding to the expected size (659 bp). This new cloning vector, named pELMO, lacked additional unnecessary DNA contained in the pDONR-221 *Pst*I-*Eco*RI region of a previously engineered pUC18*ccdB* vector (Weibel et al. 2013). This resulted in an optimised cloning vector (3306 bp) (Fig. 1). The resulting plasmid containing the *ccdB* gene was transformed into One Shot *ccdB* Survival cells and plated on LB plates with ampicillin. As expected, numerous resistant colonies were found bearing the vector. This indicated that pELMO can be propagated in *E. coli ccdB* resistant strains (Bernard and Couturier 1992) and verified the plasmid’s ampicillin resistance. Conversely, no colonies were found when pELMO was transformed in One Shot TOP10 chemically competent *E. coli* cells, thereby confirming constructed plasmid lethality.

### pELMO transformation efficiency regarding PCR products

**Fig. 2:**
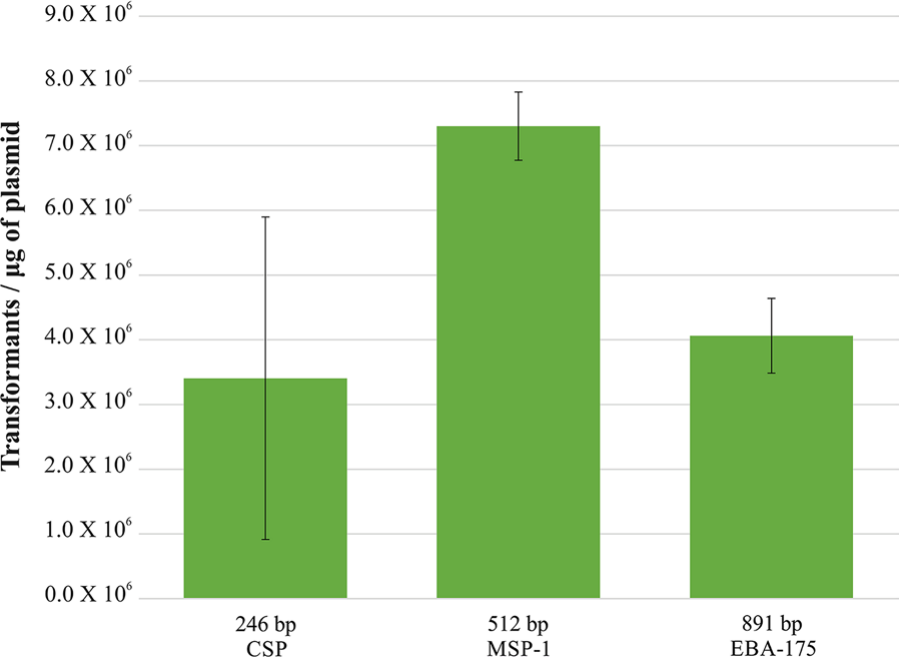
Bacteria transformation efficiency for different sized inserts. Transformation efficiency (number of transformants/µg of plasmid) for low, medium and large sized amplification products. *Solid lines* represent average values and standard deviations for two separate experiments

PCR fragments from the *P. falciparum* CSP, MSP-1 and EBA-175 protein encoding regions (246, 512 and 891 bp, respectively) were cloned into the pELMO *Sma*I restriction site and transformed into One Shot TOP10 chemically competent cells. These genes’ transformation efficiency was accurately quantified (Fig. 2). Colony PCR showed that the screened transformants were positive by amplification; however, few colonies having an unusual morphology were observed and did not have the expected fragment size by colony PCR (data not shown).

### Comparing PCR product cloning efficiency

**Fig. 3:**
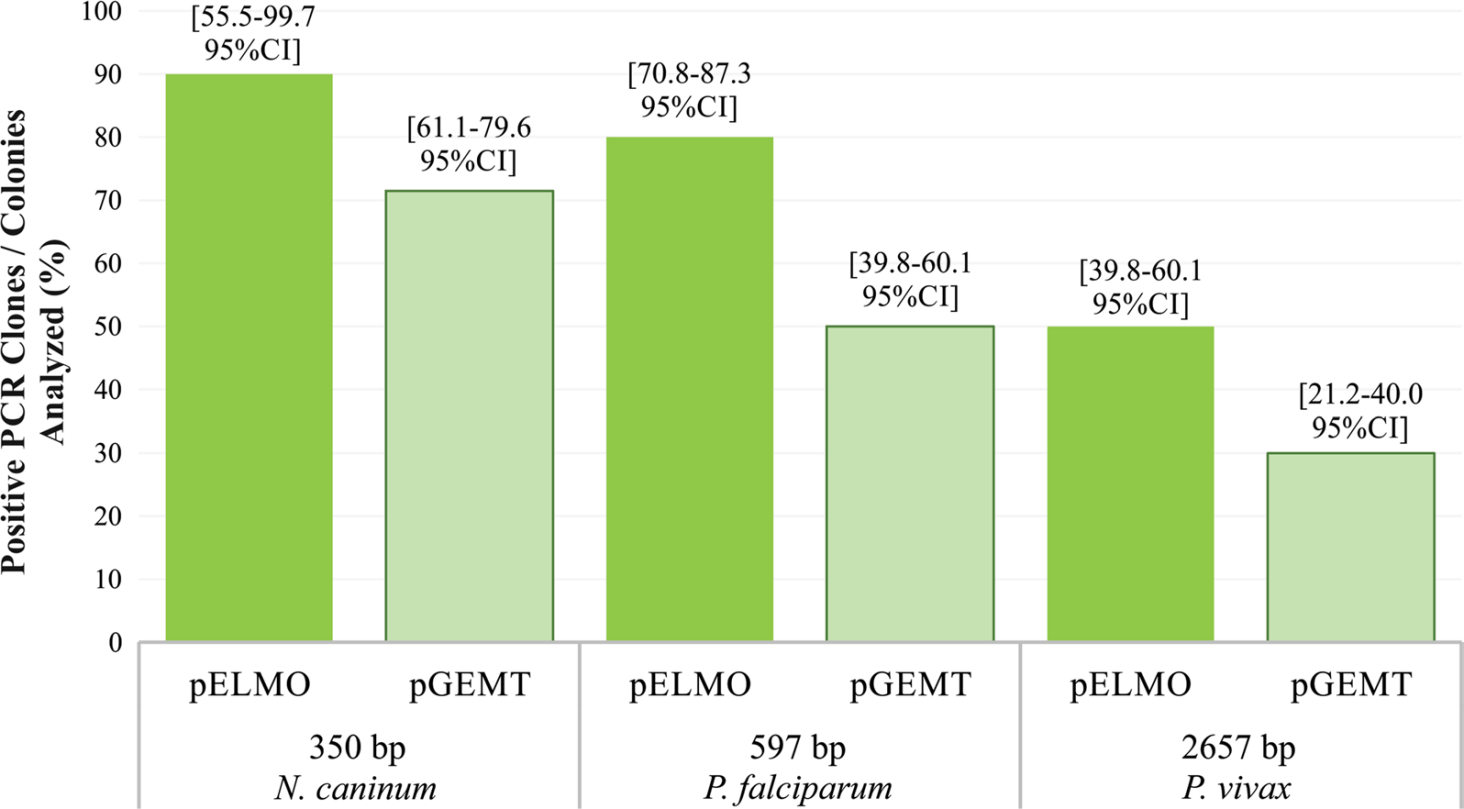
pELMO and pGEM-T Easy vector cloning efficiency for different sized inserts. pELMO and pGEM-T Easy cloning efficiency regarding low, medium and large sized inserts. Cloning efficiency is expressed as the ratio of the amount of PCR positive colonies

pELMO and pGEM-T Easy vector cloning efficiency was compared by inserting *N. caninum nc5* (350 bp) and *P. vivax arnp* (597 bp) genes into both plasmids. Statistical analysis showed pELMO cloning efficiency to be 90% (55–99 95% CI) regarding a 350 bp PCR product and 80% (70–87 95% CI) for a 597 bp PCR product. Still lower cloning efficiency was observed for the latter fragments when cloned into pGEM-T Easy vector. Only 71% (61–79 95% CI) of colonies contained a recombinant plasmid for a 350 bp DNA fragment and 50% (39–60% 95% CI) for a 597 bp fragment (Fig. 3). Mean values revealed no statistically significant differences.

The *P. vivax ron4* gene’s entire encoding sequence (2657 bp) was also used for observing how both vectors might behave when large-sized fragments were cloned into them; low recombinant colony frequency was found for both cloning systems: 50% (39–60 95% CI) cloning efficiency was estimated by pELMO and 30% (21–40 95% CI) by pGEM-T Easy vector (Fig. 3). Assays measuring cloning efficiency when using 1.5 and 2.3 kb fragment size encoding *P. vivax* proteins gave similar results (data not shown).

Plasmid inserts were then sequenced and compared to their respective reference sequence to verify the identity of *nc5*, *arnp* and *ron4* cloned fragments, as well as the efficacy of the sequencing primers for the pELMO vector (ccdBsec-F and ccdBsec-R) compared to those for the pGEM-T Easy vector (SP6 and T7). Sequencing identified around 70% of cloned insert for *nc5* and *arnp* genes in both recombinant plasmids. However, only 30% of the total insert length was identified regarding *ron4* for both pGEM-T and pELMO vectors. This may have been an effect of fragment length which could not be sequenced because it went beyond Sanger sequencing capability. The findings highlighted the fact that the pELMO cloning system performed as well as its pGEM-T Easy vector counterpart regarding insert identification through the Sanger method.

## Discussion

Vectors have been developed having numerous selection systems, i.e. traditional antibiotic-resistance markers to using toxic genes for positive transformant selection (Ainsa et al. 1996; Herrero et al. 1990; Ma et al. 2014). Positive selection systems have been designed to allow only the growth of recombinant colonies without any background on a selective medium plate (Choi et al. 2002). This kind of vector relies on lethal genes, lethal sites, dominant functions conferring cell sensitivity on metabolites or repressors of antibiotic resistance to exert such selection pressure (Bernard 1995). Several positive selection system-based vectors have been considered efficient tools to date for simplifying in vitro DNA recombination procedures (Liu et al. 2011). The *ccdB* killing gene has been widely used in constructing positive selection vectors as it has become a highly efficient lethal selection gene for DNA cloning (Weibel et al. 2013).

The pELMO vector (3306 bp) was constructed for the efficient and reliable cloning of PCR products; it contains two selection systems: the most common ampicillin-resistance marker generally used in basic research groups and the *ccdB* gene encoding a 101 amino acid toxic protein expressed by the *lac* promoter. This latter mechanism allows direct selection of positive recombinants by disrupting lethal genes, as shown in similarly constructed vectors (Bernard 1995; Gabant et al. 1997). *ccdB* expression thus results in the death of cells containing a non-recombinant vector, offering a highly efficient, positive selection system, even being comparable with white/blue selection systems based on the *LacZ* operon, one of the most used for this purpose (Bernard 1996; Messing et al. 1977). A primer set was designed (ccdBSec-Dir/ccdBSec-Rev) for sequencing plasmid inserts. pELMO digestion with the *Sma*I enzyme produced a blunt-ended vector; this bypassed the A-tailing reaction step for PCR fragments obtained by high fidelity polymerases. Such step is essential for plasmids having T-overhangs, like pGEM-T Easy Vector Systems (Promega Corporation MD, USA).

The results indicated that pELMO is suitable for cloning in the *E. coli* TOP10 strain as it does not carry the *lacI* ^*q*^ repressor, therefore granting constitutive expression of *ccdB* product without the need for IPTG (isopropyl-β-d-thiogalactopyranoside) induction. This strain lacks the F plasmid which encodes the CCDA protein; this product acts as inhibitor of *ccdB* function (Van Melderen et al. 1994).

pELMO vector transformation efficiency was ascertained by cloning *csp*, *msp1* and *eba*-*175* PCR products through blunt-end ligation into pELMO. The growth of numerous transformed TOP10 *E. coli* cells per µg DNA was seen, suggesting that *ccdB* inactivation by small insertions was highly efficient. The few colonies having unusual morphology observed in LB-amp plates (as described in other work) might have been related to the high amounts of transformant product being plated. This could have promoted local ampicillin degradation favouring satellite colony growth (Bernard 1995); less than 80 μL transformation product per Petri plate has been recommended to overcome such inconvenience. Sequencing PCR-negative transformed clones gave low-sized fragment (<60 bp) incorporation into the vector. DNA contamination might thus have been a cause for the interruption of *ccdB* toxic activity regarding these colonies, as reported previously (Weibel et al. 2013), hence reducing recombinant colony yield for the fragment being studied. Likewise, recovering clones without the insert’s sequence coincides with numerous cases where plasmid vectors, having positive selection systems, have presented several recombinant clones lacking the insert (Ma et al. 2014; Pierce et al. 1992). Some selected clones lacking the insert can often be found from impurities produced by restriction endonucleases or unspecific amplification products further ligated into the vector. PCR product purification is thus mandatory to avoid primer-dimers and other non-specific products (Bolchi et al. 2005).

The pELMO vector had higher *nc5* (350 bp) and *arnp* (597 bp) DNA fragment cloning efficiency, but not for longer fragments (>1.5 kb). Lower cloning efficiency for longer inserts shown by both vectors in cloning procedures could have been related to the fact that smaller inserts were cloned more efficiently than longer inserts (Litterer 2009). Although a 1:3 vector-to-insert molar ratio was tested to improve the recovery of clones having longer inserts (as recommended for optimal blunt fragment ligation (Rapid DNA Ligation Kit, Thermo Scientific)) as well every ligation regardless of TA vector, such strategy did not improve cloning efficiency in either of the two vectors evaluated here. The forgoing supports the idea that insert size affects a commercial vector in the same way as pELMO.

pGEM-T efficiency could also be improved by using distinct polymerases which directly incorporate adenine residues during amplification (Rittie and Perbal 2008); however, it has been reported in the literature that its use does not improve cloning efficiency in TA cloning vectors (Holton and Graham 1991; Zhang and Tandon 2012). On the other hand, a high fidelity enzyme (such as Kapa HiFi DNA polymerase) was used due to the need for obtaining amplified products identical to template. High fidelity enzymes particularly have 3′–5′ proofreading activity that affects added dATP residue stability (Sambrook et al. 1978); the resulting PCR product will thus have blunt ends. This is why this study had an additional step involving the addition of dATPs, using *taq* DNA polymerase which catalyses the non-template directed addition of an adenine residue to the 3′-end of both strands of DNA molecules to enable TA cloning in pGEM-T Easy vector.

Lower cloning efficiencies regarding all evaluated PCR products into the pGEM-T Easy system might have been due to ligation failure, given the plausible degradation of T-overhangs. This could have led to plasmid re-circularisation or the insertion of low-weight DNA fragments (Gu and Ye 2011; Oster and Phillips 2011). High-efficiency competent cells should be used when transforming; a higher sample of transformants should thus be obtained and current recombinant clone proportions could be confirmed. A major pGEM-T Easy-related selection system drawback is the growth of false-positive colonies (white colonies without the insert) and false-negative colonies (blue ones with the insert recombined). pGEM-T Easy vector cloning efficiency may thus be under-estimated as false negative colonies may result from unexpected PCR fragments cloned in-frame within the *lacZ* gene when cloning DNA fragments up to 2 kb long for these plasmids (Robles and Doers 1994). Such fragments are usually a 3 bp long multiples (including the 3′-A overhangs) and lack in-frame stop codons. False negatives may have affected cloning efficiency as only white pGEM-T Easy transformants were screened by colony PCR. Further analysis of recombinant clones indicated that sequence length was similar to that for pGEM-T Easy vector; inserts ranging from 350 to 3000 bp being efficiently sequenced. The foregoing suggests similar sequencing performance for both systems.

The modification of a previously-reported methodology for obtaining an optimised vector system (Weibel et al. 2013) has been described here. pELMO was thus selected as an alternative choice for simplifying cloning and avoiding the occurrence of clones lacking inserts; this newly designed in-house vector is a versatile efficient tool which is suitable for cloning blunt-ended PCR-products as it relies on the effect of a toxic gene from the pDONR221 sequence. Cloning PCR products in the pELMO vector thus has several advantages, offering up to 90% recombinant transformants under positive selection. When compared to commercial vectors, such as pGEM-T Easy, pELMO had more efficient cloning performance at lower cost, being easily propagated in large quantities when transforming cells in gyrA462 or lacI^q^ *E. coli* strains (the latter lacking F′ episome), without special reagents or equipment. The in-house cloning vector thus constitutes a cheap and easy-to-use choice for general cloning and sequencing procedures.

## Abbreviations

*ccdB*: gene encoding the *CcdB* protein
IPTG: isopropyl-β-d-thiogalactopyranoside
LB: Luria–Bertani
Amp: ampicillin
MCS: multiple cloning site
PCR: polymerase chain reaction
CFU: colony-forming units
bp: base pair(s)
kb: kilobase(s) or 1000 bp
CI95%: 95% confidence interval
SD: standard deviation
